# Extraction of Vanillin Following Bioconversion of Rice Straw and Its Optimization by Response Surface Methodology

**DOI:** 10.3390/molecules25246031

**Published:** 2020-12-19

**Authors:** Irnia Nurika, Sri Suhartini, Nurul Azizah, Guy C. Barker

**Affiliations:** 1 Department of Agroindustrial Technology, Faculty of Agricultural Technology, Universitas Brawijaya, Malang 65145, Indonesia; ssuhartini@ub.ac.id (S.S.); nazizah50@gmail.com (N.A.); 2 School of Life Sciences, University of Warwick, Coventry CV4 7AL, UK; Guy.Barker@warwick.ac.uk

**Keywords:** vanillin, lignocellulose, rice straw, extraction, *Serpula lacrymans*

## Abstract

Value-added chemicals, including phenolic compounds, can be generated through lignocellulosic biomass conversion via either biological or chemical pretreatment. Currently vanillin is one of the most valuable of these products that has been shown to be extractable on an industrial scale. This study demonstrates the potential of using rice straw inoculated with *Serpula lacrymans*, which produced a mixture of high value bio-based compounds including vanillin. Key extraction conditions were identified to be the volume of solvent used and extraction time, which were optimized using response surface methodology (RSM). The vanillin compounds extracted from rice straw solid state fermentation (SSF) was confirmed through LC-ESI MS/MS in selective ion mode. The optimum concentration and yield differed depending on the solvent, which was predicted using 60 mL ethyl acetate for 160 min were 0.408% and 3.957 μg g^−1^ respectively. In comparison, when ethanol was used, the highest concentration and yields of vanillin were 0.165% and 2.596 μg g^−1^. These were achieved using 40 mL of solvent, and extraction time increased to 248 min. The results confirm that fungal conversion of rice straw to vanillin could consequently offer a cost-effect alternative to other modes of production.

## 1. Introduction

Rice straw is an agricultural waste that contains high amounts of lignocellulose comprised of cellulose (32.1%), hemicellulose (24%) and lignin (18%) [1]. Cellulose and hemicellulose are both polysaccharides made up of different sugars, while lignin is an aromatic polymer synthesized from phenylpropanoid units [2]. Compared to cellulose and hemicellulose, the lignin content in rice straw is relatively low. However, since lignin is composed of abundant aromatic compounds, the effectiveness of its utilization and conversion can enhance the development of the biorefinery concept and economic growth using a circular economic approach [3,4]. Lignin is a three-dimensional biopolymer [5], that is amorphous and has three major components, *p*-coumaryl, coniferyl and synapyl alcohol as well as low molecular weight compounds such as phenols (syringols, guaiacols, catechols), aldehydes (syringaldehyde, vanillin) and phenolic ketones (acetoguaiacone, acetosyringone) [6]. Lignin therefore has potential as an important source of many valuable compounds [7], even though the depolymerization of lignin is complicated by its structure, stability and separation methods. Depolymerization of lignin using thermochemical decomposition has been shown to produce a range of low molecular weight compound, one of which is vanillin [8]. A study of vanillin production from *Jatropha curcas* showed the possibility of utilizing lignocellulose biomass for the production of vanillin [9]. The production of vanillin using bioconversion of certain natural substances such as lignin, ferulic acid, eugenol, isoeugenol, etc., using microorganisms such as yeast, fungi, and bacteria [10] has been suggested for use as a flavour compound [11] for the food, beverage and aroma industries [12,13].

The use of fungi to convert biomass offers a novel but cost-effective route for the production of high value chemicals such as vanillin. There are two main groups of fungi that have evolved mechanisms capable of lignocellulosic conversion, which are the white rots and brown rots. In contrast to the white rots which target the lignin, the brown rots preferentially degrade cellulose and hemicellulose while the lignin is mostly left untouched. *Serpula lacrymans* is considered as one of the most aggressive brown rot fungi [14] yet this fungus does not contain the ligninolytic enzymes which the white rots use to modify lignin. *S. lacrymans* produces quinone and oxalic acid compounds that are involved in the generation of Fenton reactions which produce hydroxyl radicals (OH∙) [15,16]. This Fenton chemistry is thought to play a key role in degrading lignocellulose [14,17]. The breakdown of lignocellulose by such fungi is however not specific and results in a mixture of different compounds. To be useful these compounds need to be first extracted then separated. The solvents used are important variables that influence the efficiency of the extraction process [18]. In addition, because metabolites diffuse during culture, long extraction times may be needed for complete product recovery [19]. The use of methanol, ethanol, acetone and distilled water solvents have been compared for extraction of antioxidant and phenolic compounds present in biomass and the results showed that ethanol was efficient [18,20,21]. Dong et al. [22], demonstrated ethanol can dissolve vanillin compounds and improve the extraction of vanillin from *Vanilla planifolia*. Ethyl acetate also has a high affinity for vanillin [23,24,25,26]. In addition to the type of solvent, the solubility of vanillin in the extraction process is influenced by the extraction time [27]. The quantity of the extracted material is improved due to increasing contact between the material and the solvent which can increase the saturation point of the solution [28]. The longer the extraction time, the higher the percentage of vanillin that is collected [22]. Other techniques including ultrasound-assisted extraction (UAE), pressure-assisted extraction (PAE), microwave-assisted extraction (MAE) and maceration can be used to increase the yield of vanillin [22]. The variation in approaches and differences in yield of vanillin obtained suggest that optimization of extraction techniques is required. Response surface methodology (RSM) is an important tool use for statistically designing experiments and building models which is used to examine the effect among numerous factors at diverse levels. In this study, RSM was applied in order to investigate the effect of solvent volumes and extraction times to predict the maximum concentration and yield of vanillin produced from rice straw which utilised biomass conversion by the brown rot fungus *Serpula lacrymans*. Currently the commercial production of natural vanillin, supplies less than 1% of the total market demand [29,30]. The demonstration that vanillin can be achieved through solid state fermentation (SSF) of biomass is not sufficient, therefore it is important to also optimise its subsequent extraction. This paper highlights the potential use of *Serpula lacrymans* for depolymerization of lignocellulose biomass feedstock to generate the production of vanillin and shows how optimization of vanillin extraction conditions can improve the process and hence is a great step forward to making it economically viable.

## 2. Results and Discussion

### 2.1. The Changes of Total Soluble Phenols, Total Reducing Sugars, pH and Weight Loss during Rice Straw SSF

**Figure 1:**
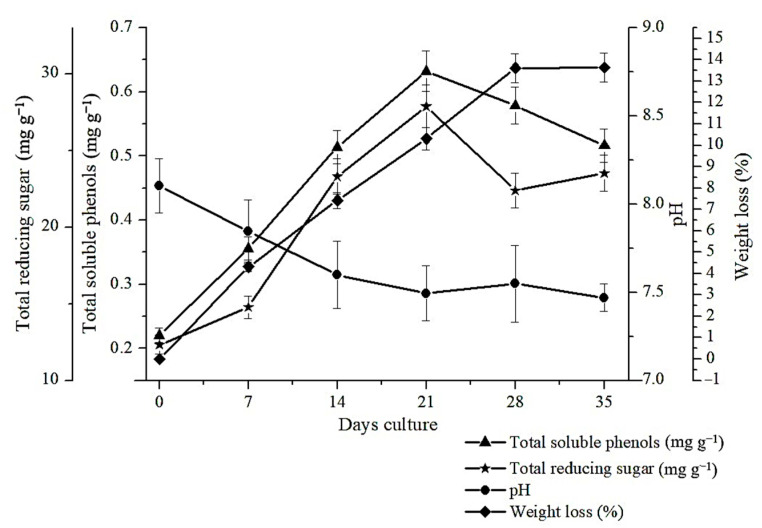
The relationship between total reducing sugar (mg g^−1^), total soluble phenols (mg g^−1^), pH and weight loss (%) of the feedstocks rice straw inoculated using *Serpula lacrymans* for 35 days. The error bar represent the least significant different (LSD 5%) derived from ANOVA analysis.

Within the aqueous extracts of rice straw inoculated with *S. lacrymans,* the total soluble phenols were observed to increase gradually from day 0 (0.220 mg g^−1^) to day 21 (0.632 mg g^−1^) then to decline (Figure 1). These results suggest that the amount of total soluble phenols produced is time dependent. It has previously been reported that the time taken for lignocellulose degradation is substantially greater using microbial approaches compared to other methods such as chemicals or high temperature pretreatments [31]. It is therefore unsurprising that the number of aromatic compounds released increased with time [32]. The brown rot fungi produce oxalic acids and hydroxyquinones that reduce iron and generate Fenton reactions [33] which subsequently released hydroxyl radicals (OH•) to modify lignocellulose. According to Arantes et al. [17], phenols can also play a role in the Fenton reaction and in the reduction of Fe^3+^ to Fe^2+^. The longer-term decline in total phenolics detected, might reflect their consumption in such processes. 

*S. lacrymans* has also been shown to be capable of decomposing cellulose and hemicellulose [34] although this is hard to be observed as the total reducing sugar fluctuates. This is expected as the sugars are consumed as a source of nutrition for fungal growth. The total reducing sugars detected in the extracts increased linearly up to day 21 and then fluctuated (Figure 1). The highest amounts of sugars were extracted on 21 days (27.87 mg g^−1^). This is thought to be due to the amount of sugars released being higher than the sugars consumed by the fungi, and might correlate with the enzyme activity in reducing lignocellulose [35]. According to Tengerdy and Szakacs [36], in pretreatment, sugar is produced through the metabolism of microorganisms. The fungus breaks down the cell wall and then converts cellulose and hemicellulose into soluble material [35]. The subsequent decrease in total reducing sugar is thought to be due the fungus utilising the glucose content in the material is as a carbon source for its own metabolism [37], but an alternative theory is that the production of toxic phenols could inhibits the process of cellulose breakdown [38].

Changes in pH (Figure 1) indicate the production of organic acids such as oxalic acid by fungi during the degradation process [17,39,40,41]. At the beginning of incubation, the pH is high which can be attributed to the decomposition of organic nitrogen which releases ammonia [42]. During the incubation period, there is a decrease in pH due to depletion of organic nitrogen, which is easily degraded. Oxalic acid is one of the dominant organic acid produced at initial incubation phase of fungi, which is generally produced when the pH value above 6.0 [41,43]. The brown rot fungi have two enzymes that can produce oxalic acid, glyoxylate dehydrogenase and oxaloacetate hydrolase [44]. The releasing of oxalic acid by the brown rot fungi can stimulate the generation of OH radicals under Fenton reaction, which contributed on lignocellulose breakdown [45]. Weight loss was observed between day 0 to day 28, after which little further degradation occurred, which supports the theory that the process is inhibited when phenolic breakdown products reach a certain level. The weight loss reflects the fungi’s action which causes lignocellulose degradation through damage to the cell walls [46] and the subsequent release of sugars and aromatic compounds [47]. The releasing sugars in the form of mono and oligosaccharides were then used as sources of carbon and energy. Moisture loss through evaporation is also a possibility in accounting for weight loss [48]. This was counteracted through the jars used and its impact is thought to be minimal. 

**Figure 2:**
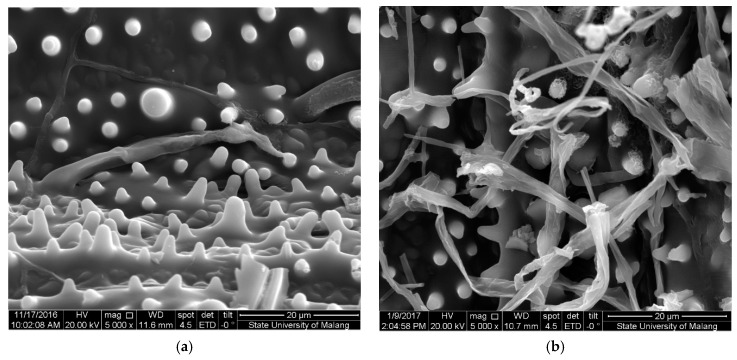
SEM images of untreated rice straw 0 days incubation (**a**) and pretreated rice straw for 35 days incubation (**b**) with a magnification of 5000×.

Pretreatment of rice straw will also cause changes in its surface structure. Surface analysis using scanning electron microscopy (SEM) on rice straw before and after pretreatment with *S. lacrymans* is shown in Figure 2. Figure 2a shows no damage to the rice straw surface before pretreatment, the structure is rigid and very dense. According to Mukherjee et al. [49], rice straw surface appears to maintain structural uniformity without any distortion in the alignment of the fibers.

Rice straw surface after pretreatment with 35 days incubation (Figure 2b) shows the surface is cracked and the structure are not solid. According to Nurika et al. [50], changes in the surface of rice straw indicate that biodegradation affects the surface area of rice straw and causes the opening of the holo-cellulose fibrils.

### 2.2. Optimization of Pretreated Variables

In addition to knowing when to harvest for optimal yield, optimization of the extraction process is also important. This was carried out using a centralized composite design. For this, the amount of solvent and extraction time were selected based on the sample that produced the highest yield of phenols from the results above (Figure 1), which was found to be released following 21 days of incubation and therefore this time point was used to optimize the extraction. Both the extraction time and solvent volume affected the response of vanillin concentration and yield. The combination of treatments and responses can be seen in Table 1 and Table 2.

### 2.3. Effect of Different Solvent on Vanillin Extraction

#### 2.3.1. Ethanol

The regression coefficients for the models used for vanillin concentration (Table 3) and the ANOVA results using ethanol as the solvent are presented (Table 4). The quadratic models proved statistically significant with a *p*-value of 0.0002, the lack of fit value also proved to be not significant (*p*-value 0.59). The R^2^ value is 0.95 which indicates that the volume of ethanol and extraction time are responsible for 95% extraction of the vanillin content, and the remaining 5% is influenced by other factors such as the type of solvent and temperature. According to Shakeel et al. [26], several types of environmentally friendly solvents such as transcutol, propylene glycol (PG), and polyethylene glycol-400 (PEG-400) can dissolve some organic compounds such as vanillin. 

Similarly, the model for vanillin yield also showed a significant *p*-value of 0.0001 and a lack of fit value of 0.4041, indicating that this model is also appropriate. The R^2^ value of 0.96 indicates that as with vanillin concentration, the ethanol volume and extraction time affect the vanillin yield of 96%, and the remaining 4% is influenced by other factors.

**Figure 3:**
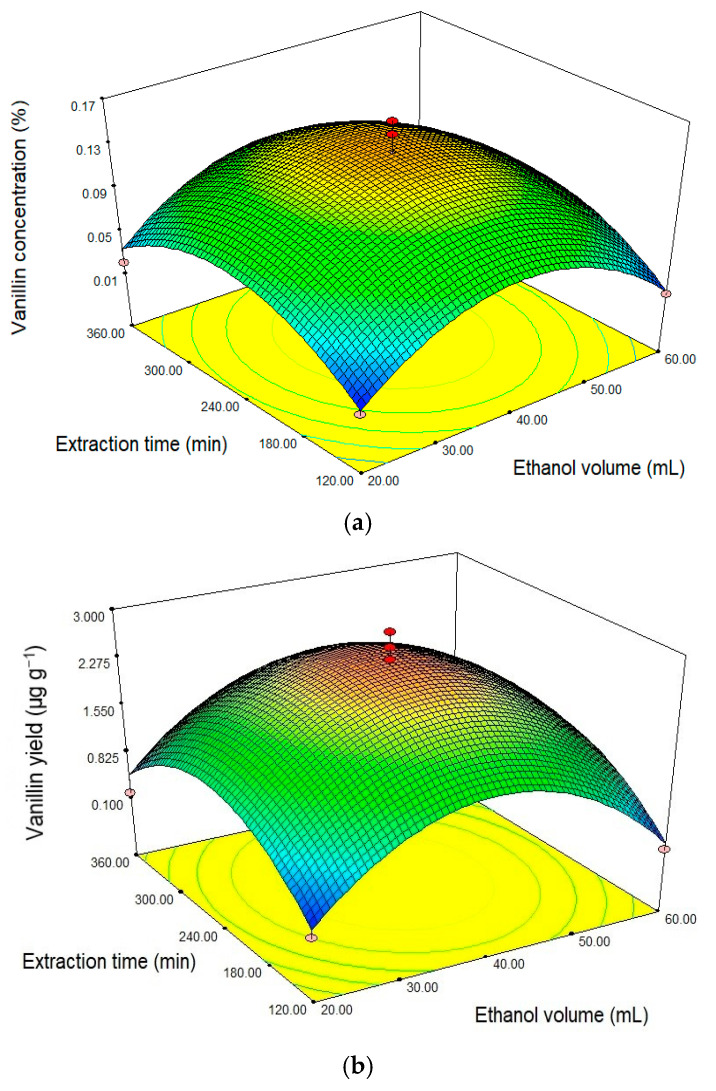
Response surface curve (the 3D plots) showing the relationship between ethanol volume and time extraction toward the response of: (**a**) vanillin concentration (%) dan (**b**) vanillin yield (μg g^−1^).

Figure 3 shows the effect of ethanol volume and extraction time on vanillin concentration and yield. Vanillin concentration increase with the ethanol volume until the extraction time reaches a certain point (Figure 3a), after this the percentage of vanillin decreases if either the volume of ethanol or the lengths of extraction are increased. The highest response rate was obtained using 40 mL solvent and 240 min extraction time with a response value of 0.16%, and after this point the response value decreased to 0.116% (Table 1). Dong et al. [22] reported that higher volume of solvents will increase the amount of vanillin obtained due to mass transfer and increasing extraction efficiency. This study shows that the extraction time also affects the percentage of vanillin obtained at the optimum limit. Beyond this point the solvent will evaporate with if the extraction time is increased further and therefore the solvent ability to reach the solute is reduced [22]. One advantage of using ethanol is that it can be used as an organic solvent to avoid the use of more toxic solvents such as methanol, acetonitrile, chloroform or hexane [51]. 

Vanillin yield also increases with increasing ethanol volume and extraction time until a critical point is reached (Figure 3b), beyond this it decreases if either the ethanol volume or extraction time is increased. The vanillin yield increases as the volume of solvent increases until the optimum limit is achieved. This is likely due to increasing contact between materials and solvents which facilitates mass transfer and increases the extraction efficiency [52].

The extraction time also affects the yield of vanillin obtained. Increasing extraction time can significantly accelerate the movement of molecules, which also means that solvents have a longer time to enter between the matrix to dissolve the structure so that compounds become more accessible [22]. The highest yield response was predicted to be with 40 mL solvent volume and 240 min extraction time with a response value of 2.995 µg g^−1^ (Table 1).

#### 2.3.2. Ethyl Acetate 

The regression coefficients for the ethyl acetate models used (Table 5) and the associated ANOVA results are shown in Table 6. The quadratic models show a significant effect on vanillin levels (*p*-value < 0.0001). Lack of fit testing also proved to be not significant (*p*-value is 0.6214). An R^2^ of 0.98 indicated that the volume of ethyl acetate and extraction time accounted has the greatest effect on vanillin concentration while the remaining 2.36% is influenced by other factors not included in the model. 

The model also works for vanillin yield, (*p*-value < 0,0001). However, a lack of fit value (of 0.0791) indicates that the model was very different from the model found using ethanol. The R^2^ value of 0.99 indicates that ethanol volume and extraction time affect the vanillin yield of 99.08%, and the remaining 0.92% is influenced by other factors that were not included in the model.

**Figure 4:**
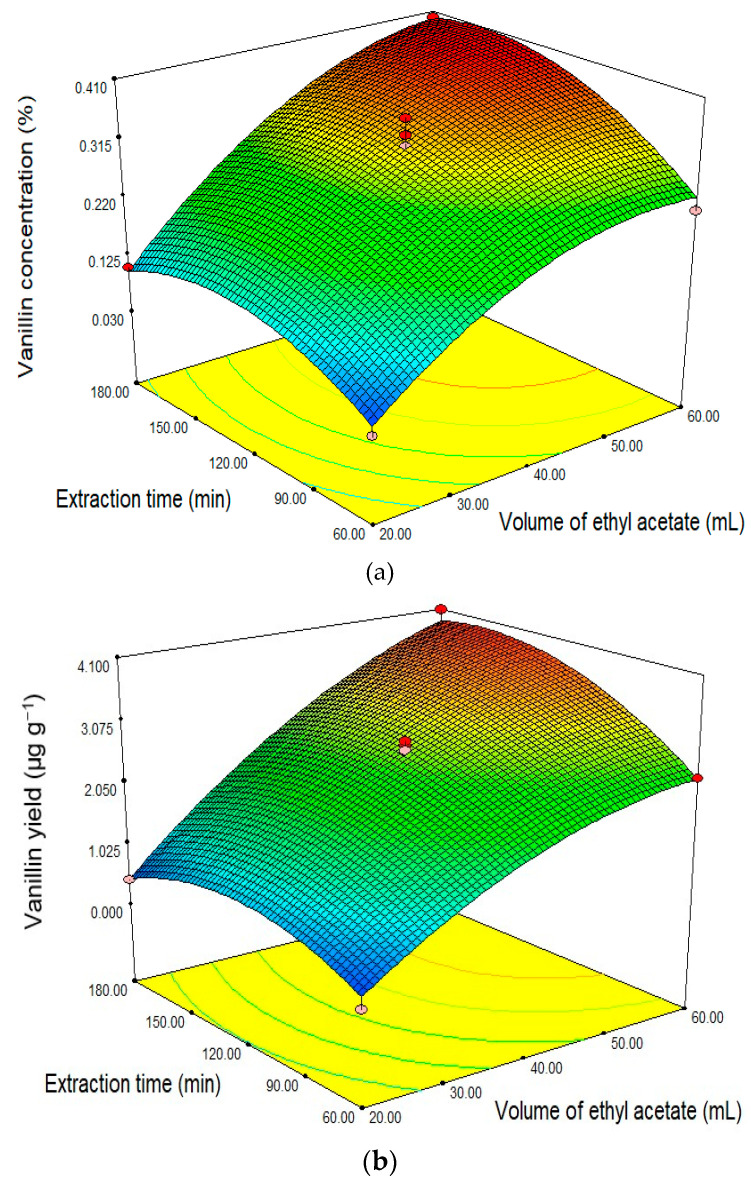
Response surface curve (the 3D plots) showing the relationship between ethyl acetate volume and time extraction toward the response of: (**a**) vanillin concentration (%) and (**b**) vanillin yield (μg g^−1^).

The effect of ethyl acetate and time of extraction on vanillin concentration and vanillin yield as modelled is shown in Figure 4. There was a striking difference in these models compared to those generated for ethanol (Figure 3). Figure 4a shows an increase in vanillin levels as the volume of ethyl acetate and/or the extraction time is increased up to a point but then decrease with a further increase in the volume of ethyl acetate and extraction times. In this study, the highest response rate of vanillin concentration was obtained using 60 mL of ethyl acetate and 180 min time of extraction with a response value of 0.399% (Table 2). This suggests that more ethyl acetate will be required compared to ethanol. The solvent volume used in the extraction process is critical for the economic production of vanillin [53]. The amount of material and the volume of solvent used also has a significant effect on the extraction results of vanillin compounds [54] as does the duration of the extraction process also affects the extraction results obtained [55]. Jadhav et al. [56], suggests that the longer the extraction the greater the vanillin yield obtained, but the vanillin yield decreases beyond the optimal point due to the solvent becoming saturated.

The model of vanillin yield (Figure 4b) also shows the vanillin yield increases with increasing volume of ethyl acetate and extraction time. The highest vanillin yield response was obtained at 60 mL solvent volume and extraction time 180 min with a response value of 4.077 µg g^−1^. Further extraction beyond this time appears to have a marginal effect on the yield of vanillin. Research by Jadhav et al. [56], also showed that the ratio of the volume of solvents and materials had a significant effect on the yield of vanillin. The current work suggests that optimization of time and solvent used can be critical for achieving cost efficiencies.

### 2.4. Optimization Concentrations and Yield of Vanillin Extract

The factor combination approach predicts very different results when the two solvents were compared (Table 7 and Table 8). The optimal solutions derived for each solvent show that both the amount of solvent used and the time taken differ and can impact the yield of vanillin. Ethyl acetate may extract nearly 35% more vanillin compared to the ethanol. The results of the optimal solution have prediction of vanillin levels obtained at 0.408% and vanillin yield of 3.957 μg g^−1^ with a desirability function of 0.985 (Table 8). The ethyl acetate extractions were achieved approximately 33% quicker than the ethanol extractions. According to Montgomery [57], the desirability function should be used to determine the degree of accuracy of the results of the optimal solution. The closer the desirability indexes to the value of 1, the higher the accuracy of the optimization which indicates that ethyl acetate is the better solvent, as its desirability index was 0.985.

Numerous studies have investigated vanillin production from ferulic acid, eugenol and lignin using microorganism as reviewed by Kaur and Chakraborty [58]. The main issue is the toxicity of vanillin and other phenols to microorganisms consequently efficient extraction is important. The low yield is thought to be due to the extraction method used. This study used a conventional extraction method in the form of maceration with shaking using a waterbath shaker. Improvements such as microwave extraction could be applied in conjunction with optimised solvent use could be used to increase the efficiency of extraction. 

### 2.5. Confirmation of Vanillin Content within Extracts

**Figure 5:**
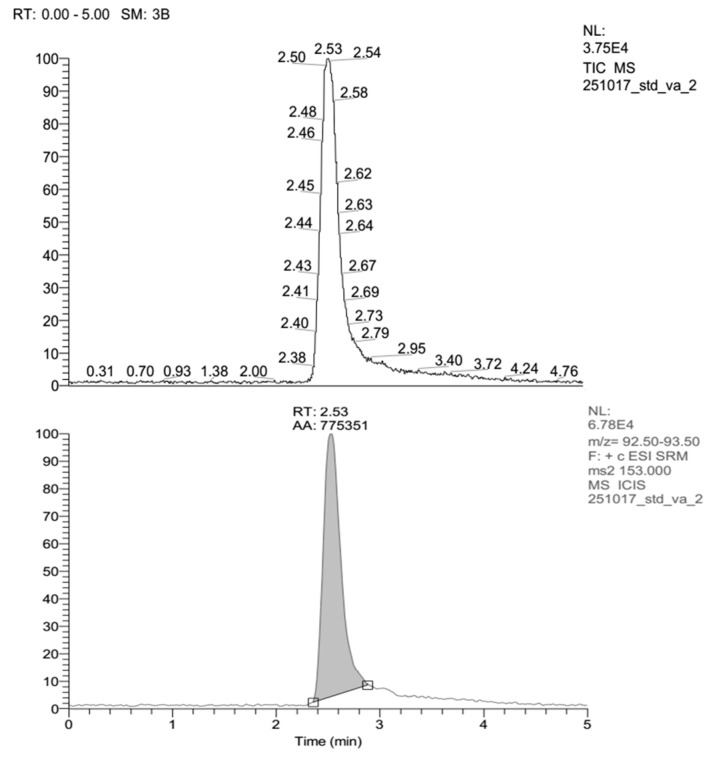
Chromatogram LC-ESI-MS/MS showing vanillin standard (ion precursor and ion product).

Vanillin is an aromatic aldehyde (3-methoxy-4-hydroxybenzaldehyde), including a group of simple phenolic compounds with formula C_8_H_8_O_3_ which are functional groups of aldehydes, ethers and phenols or phenols connected with aldehydes and methoxy groups [59]. The compounds produced following the optimal extraction approach developed above were subsequently identified using LC-ESI-MS/MS. Vanillin has a molecular weight of 152.149 g/mol. Therefore, the identification of vanillin LC-ESI-MS/MS was assayed on molecular weights between 152–153 g/mol. Figure 5 shows the chromatogram of precursor ions and standard vanillin product ions. The retention time (RT) of the standard vanillin product is 2.53 min based on its ion chromatogram.

**Figure 6:**
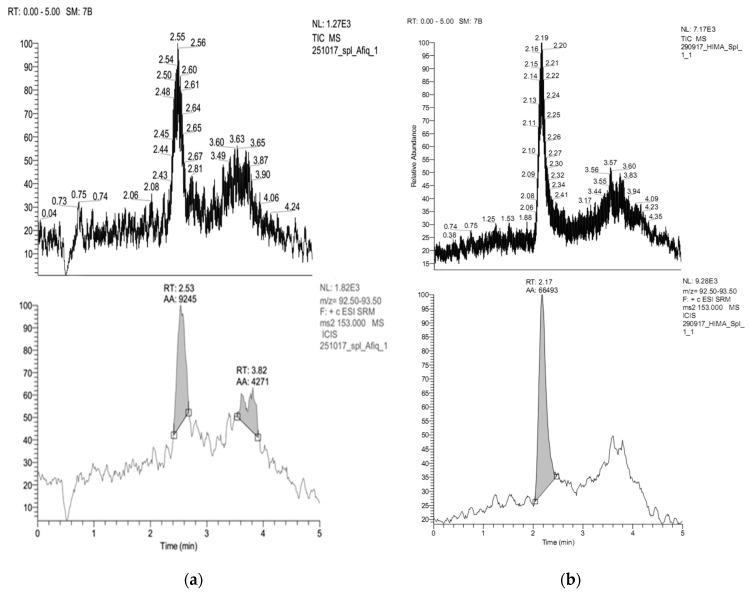
Chromatogram LC-ESI-MS/MS from the extraction of rice straw using: (**a**) ethanol; and (**b**) ethyl acetate.

The chromatogram from LC-ESI-MS/MS of the sample extracted from rice straw with ethanol solvent is shown in Figure 6a. The product ion shows the RT is 2.53 min, which is the same as the RT of the vanillin standard. According to Kaur and Chakraborty [58], the vanillin content in rice straw based on dry weight is about 0.012%, and based on the weight of the sample which is chemically degraded, the vanillin content in rice straw is around 0.009%.

Analysis of vanillin compound from samples extracted from rice straw with ethyl acetate can be seen in Figure 6b. Peak points adjusted to vanillin standard conditions showed RT values of 2.17 min. The vanillin identification results showed slightly faster retention times compared to Rana et al. [60] where vanillin retention time of 2.76 min was identified using HPLC and Dal Bello’s [61] retention time of 6.5 min. The variation in retention time can be predicted due to differences in total time of analysis and the set up used. In addition, the selection of the stationary phase and the mobile phase in the identification process is also thought to produce different retention times.

## 3. Materials and Methods 

### 3.1. Fungal Strain and Medium 

*S. lacrymans* was obtained from the collection of Bioindustry laboratory, Department of Agroindustrial Technology, University of Brawijaya, Indonesia. The culture was stored at 4 °C and grown on malt extract agar (MEA). Agar plugs of mycelia were added to barley grain and grown to produce inocula (grain spawn) at 22 ± 2 °C.

### 3.2. Solid State Fermentation (SSF) 

Freshly threshed rice straw was collected from a farm in the vicinity of the University of Brawijaya, Malang, Indonesia. Rice straw were chopped into small segments of 1–2 cm length. 10 g of rice straw was placed into honey jars and added 13 mL of water before being autoclaved twice at 121 °C for 1 h each time. The rice straw was subsequently inoculated with 1 g of grain spawn of *S.lacrymans* and incubated at the optimal temperatures (22 ± 2 °C) for 35 days. 

### 3.3. Aqueous Extraction

Aqueous extraction of rice straw after incubation with *S. lacrymans* was used to determine the amounts of total reducing sugar and soluble phenols. 150 mL of purified water was boiled to 80 °C, this was added and shaken at 100 rpm for 15 min at 40 °C in an orbital shaker. The biomass from individual jars was squeezed by hand through fine muslin netting. The solid cake was retained for subsequent solvent extraction. The aqueous extract was centrifuged at 13,000× *g* for 10 min and the liquid layer filtered through a Buchner funnel using a 7 μm glass fibre filter and frozen at −20 °C before analysis.

### 3.4. Solvents Extraction

Ethanol and ethyl acetate were used to extract vanillin and to remove other phenolic products released from the biomass samples. The extraction was conducted on the solid biomass cake post-aqueous extraction described above; this involved a number of steps. The steps were: (i) following the aqueous extraction, the liquid was carefully removed leaving a solid residue, (ii) the resulted sample cake was placed into a clean 500 mL conical flask and weighed to obtain the pre-extraction weight, (iii) solvents (ethanol and ethyl acetate) was added to the biomass cake and the sample then placed to water-bath shaker at 30 °C; 150 rpm, while the period of extraction was based on each treatment (Table 1 and Table 2) (iv). The sample was then filtered. Once all of the filtrate had passed through the filter it was transferred to a round-bottomed flask and the biomass was removed from the funnel and placed onto labeled aluminium foil. (v) The remaining biomass was dried in an oven to obtain a final post-extraction dry weight. (vi) The filtrate was dried using a vacuum rotary-evaporator (40 °C and 100 rpm) in order to precipitate out the phenolics held in the solution and to remove any remaining water. The volume of solvents (ethyl acetate and ethanol) and extraction time were varied according to the combination of treatments indicated by the Response Surface Method (RSM). 

### 3.5. Response Surface Methodology (RSM) Design

The response surface method was developed using a centralized composite design (CCD) to determine the optimal solvent volume and vanillin extraction time. Solvent volume: X_1_ (20, 40, and 60 mL) and time of extraction: X_2_ (120, 240 and 360 min) were determined as the fixed variables in order to optimize the extraction process. The extraction time factor used was based on previous research. 70% (*v*/*v*) ethanol-water extraction was used with times that varied between 120 min to 720 min [22]. The range of ethanol volumes and the extraction times were based on research using wheat straw cultures with *Rhizopus oryzae* RCK2012 [21]. The range of volume for ethyl acetate extraction was set between a lower limit value of 20 mL and an upper limit of 60 mL. The timing for extraction varied between 60 min and 180 min. These limits were based on previous studies [62,63]. The optimized response factors used were the vanillin concentration and yield.

The experimental data was interpreted with a second order response surface model with the following forms:

(1) $$y=\beta_{0}+\sum_{j=1}^{k}\beta_{j}x_{j}+\sum_{j=1}^{k}\beta_{jj}x_{j}{}^{2}+\sum^{\;}\sum_{i<j}\beta_{ij}x_{i}x_{j}$$

where *y* is the response tested, in this case in the form of concentration and yield of vanillin. *β*_0_, *β_j_*, *β_jj_*, and *β_ij_* are constant coefficients for intercept, linear, square, and interaction terms. The factors and levels of factors studied for ethanol and ethyl acetate can be seen in Table 9.

X_1_ and X_2_, represent the fixed variables (solvent volume and extraction time). Data analysis was performed using Design-Expert software (version 7.0.0., State-Ease, Inc., Minneapolis, MN, USA). Analysis of variance (ANOVA) was also used to evaluate the quality of the fit model. The test of statistical differences is based on the total error criteria using a 95.0% confidence level. 

### 3.6. Total Reducing Sugars and Soluble Phenols 

Total reducing sugar and soluble phenolics analysis were performed on the aqueous extract samples. Reducing sugars were determined colourimetrically by the DNS (dinitrosalicylic acid) method using glucose as the standard and the absorbance was read at 540 nm [64] using a spectrophotometer UV-Vis (Thermo Scientific type Genesys 10 UV, (Waltham, MA, USA). In order to minimize sugar variation, 3 replications were used for each samples.

Total soluble phenols analysis was performed on the aqueous extract samples prepared as above. The Folin-Ciocalteau method using gallic acid as the standard [65] was used to measure phenols colourimetrically. Absorbance was read at 760 nm using a spectrophotometer (UV-Vis merk Thermo Scientific type Genesys 10 UV). Total soluble phenol is expressed as milligrams of gallic acid per gram of extract (dry weight). To minimize sample variability, 3 technical replicates were analysed for each sample. 

### 3.7. Weight Loss (% Dry Weight)

Samples were taken at different times of culture (0, 7, 14, 21, 28 and 35 days). At each time point three samples were removed from each cultivation jar and these were oven-dried at 100 °C until a constant weight was reached. Weight loss was estimated as the difference between the weight of the whole culture in medium at the beginning and at the end of the pretreatment.

### 3.8. Scanning Electron Microscope (SEM)

The changes of surface structure of untreated and pretreated of OPEFB were analyzed using Scanning Electron Microscope (a NanoSEM-FEI Nova 200, type Inspect-S50, Hillsboro, Oregon< USA). Lignocellulosic rice straw SSF sample were dried and cut (approximately 1 cm length and 1 mm of thickness). The samples were then affixed to a carbon tip and placed on a coating tool (sputter coating) for vacuum and coating process (using mini sputter coater EMITECH type SC7620, Laughton, East Sussex, UK). The vacuum process was then carried out for 30 min or until the pressure was between 4 and 6 Pa and followed by a coating process for 3 min which is marked by emergence of a purple coloration. The sample was then ready to be tested using SEM. 

### 3.9. Vanillin Concentration and Yield 

The extracts were evaporated using a rotary evaporator (RV 10 control, IKA, Goteborg, Sweden) at 40 °C, 50 rpm until dry. The samples were then redisolved in 2.5 mL distilled water, and 250 μL 0.1 N NaOH. A standard solution was prepared using 0.05 g vanillin (Sigma Aldrich, Singapore, Singapore) dissolved in solution of 2.5 mL ethyl alcohol and 50 mL distilled water. 6 different amount of vanillin dilutions were prepared from this solution using 0 μL, 50 μL, 100 μL, 150 μL, 200 μL and 250 μL followed by addition of distilled water to a final volume of 2.5 mL after which 250 μL 0.1N NaOH was added. The blank was made by mixing 2 mL 0.1 N NaOH with 98 mL of distilled water. The vanillin concentration was measured according to standard protocols [54] using a UV-Vis spectrophotometer wavelength at 348 nm. The vanillin content is stated as follows:

(2) $$\mathrm{Vanillin}\;\mathrm{concentration}\;\left(\%\right)=\frac{X\times F_{p}}{M\times 10}\times 100\%$$

*X* = Sample solution concentration (ppm) obtained from the standard vanillin calibration curve
*F_p_* = Dilution factor
*M* = Sample weight (mg)

The vanillin yield [66] was subsequently calculated based on the amount of material used as follows:

(3) $$\mathrm{Vanillin}\;\mathrm{yield}\;\left({\mu g\;g}^{-1}\right)\;=\frac{\mathrm{vanillin}\;\mathrm{concentration}\;\left(\mathrm{ppm}\right)\times \mathrm{sample}\;\mathrm{volume}\;\left(\mathrm{mL}\right)}{\mathrm{Total}\;\mathrm{weight}\;\mathrm{sample}\;\left(g\right)}$$

### 3.10. Identification of Vanillin Compound Using Liquid Chromatography-Electrospray Ionization Tandem-Mass Spectrometry (Modification by Ong et al. [67])

LC-ESI MS/MS were used to confirm the vanillin content using Liquid Chromatography combined with Electrospray Ionization tandem Mass Spectrometry (LC-ESI-MS/MS). A two hundred µL sample was added to 1 mL of methanol (HPLC grade) and 100 µL Pb acetate 0.06 M. Samples were centrifuged at a speed of 3500 rpm for 5 min. The supernatant was taken and dried using an oven at a temperature of 400 °C. 3 mL of methanol was subsequently added and then filtered with a 0.2 microns polytetrafluoroethylene (PTFE) filter. The sample was tested using a Hypersil Gold column (50 mm × 2.1 mm × 1.9 µm) (Thermo Fisher Scientific, Waltham, MA, USA). UHPLC (ACCELLA type 1250 is made by Thermo Fisher Scientific). The technical specification were as follows: a) a partial loop with an injection volume of 3000 µL, b) 400 µL flush volume and speed of 100,000 µL/s. c) spraying speed of 8000 µL/s. The analysis time was ±9 min. Solvent A = 0.1% formic acid in water; and B = 0.1% formic acid in acetonitrile. The speed of gradient mobile phase was 250 µL/min and the injection volume was at 2 µL LC. The column was controlled at 30 °C, and the auto sampler compartment was set to 16 °C.

## 4. Conclusions

This study has shown that vanillin can be extracted from rice straw following culture with the fungus *S. lacrymans*. The innovative use of response surface methodology also confirmed this as an effective tool for optimization of extraction parameters. The coefficient of determination (R^2^) values for all parameters showed a good fit between the model and the experimental data, at above 95% confidence level. The results showed that operating parameters (solvent volumes and extraction times) had significant effect on concentration and yield of vanillin. The maximum concentration (0.165%) and yield vanillin (2.596 μg g^−1^) extracted using ethanol was obtained from the use of 39.86 mL solvent and 247.98 min of extraction time. While using 60 mL ethyl acetate for 159.56 min the predicted maximum concentration and yield vanillin obtained were 0.408% and 3.957 μg g^−1^ respectively. Although low concentrations and yield of vanillin were achieved during the conversion of the rice straw, the methodology proved effective. Other study has also included temperature in calculations made using RSM [68], which showed the effect of parameters such as temperature. Investigation of all such possible parameters will also need to be investigated in the future to ensure optimal extraction. Efficient extraction conditions are essential if such biomass is to be utilized in the future as a source of such valuable chemicals. This means the ability to select different solvents, as their use will be more commonly based on their environmental footprint, which can be minimized by optimizing volumes used. The demonstration of the effectiveness of such tools as RSM is therefore important in order for us to move towards more efficient use of our natural resources including our biomass. 

## Figures and Tables

**Table 1:** Response of concentration and yield of vanillin produced from lignocellulose degradation on rice straw (from treatments: volume of ethanol and extraction time).

Run	Code Variable		Actual Variable		Response	
	X_1_ (Solvent Volume)	X_2_ (Extraction Time)	Volume Ethanol (mL)	Extraction Time (min)	Vanillin Concentration (%)	Vanillin Yield (µg g^−1^)
1	−1	−1	20	120	0.014	0.121
2	1	−1	60	120	0.014	0.130
3	−1	1	20	360	0.020	0.178
4	1	1	60	360	0.018	0.163
5	−1.414	0	11.72	240	0.019	0.162
6	1.414	0	68.28	240	0.014	0.114
7	0	−1.414	40	70.29	0.033	0.685
8	0	1.414	40	409.71	0.067	1.207
9	0	0	40	240	0.124	2.412
10	0	0	40	240	0.161	2.995
11	0	0	40	240	0.116	2.190
12	0	0	40	240	0.150	2.773
13	0	0	40	240	0.132	2.596

**Table 2:** Response of concentration and yield of vanillin from lignocellulose degradation on rice straw (from treatments: volume of ethyl acetate and extraction time).

Run	Code Variable		Actual Variable		Response	
	X_1_ (Solvent Volume)	X_2_ (Extraction Time)	Volume Ethyl Acetate (mL)	Extraction Time (min)	Vanillin Concentration (%)	Vanillin Yield (µg g^−1^)
1	−1	−1	20	60.00	0.044	0.176
2	1	−1	60	60.00	0.234	2.481
3	−1	1	20	180.00	0.105	0.418
4	1	1	60	180.00	0.399	4.077
5	−1.414	0	11.72	120.00	0.032	0.075
6	1.414	0	68.28	120.00	0.387	3.722
7	0	−1.414	40	35.15	0.164	1.310
8	0	1.414	40	204.85	0.263	2.105
9	0	0	40	120.00	0.337	2.826
10	0	0	40	120.00	0.364	2.912
11	0	0	40	120.00	0.291	2.654
12	0	0	40	120.00	0.313	2.718
13	0	0	40	120.00	0.318	2.761

**Table 3:** Regression coefficient of polynomial function from all response surface models of ethanol extraction.

Term	Coefficient	Standard Error	F-Value	*p*-Value
**Vanillin Concentration**				
Intercept	0.14	0.0078		
X_1_	−0.0012	0.0062	0.036	0.8555
X_2_	0.0073	0.0062	1.39	0.2767
X_1_X_2_	−0.0007	0.0087	0.0065	0.9381
X_1_^2^	−0.064	0.0066	94.56	<0.0001
X_2_^2^	−0.047	0.0066	51.57	0.0002
R^2^ = 0.95				
**Vanillin Yield**				
Intercept	2.59	0.15		
X_1_	−0.0092	0.12	0.0063	0.9387
X_2_	0.10	0.12	0.80	0.4015
X_1_X_2_	−0.006	0.16	0.001339	0.9718
X_1_^2^	−1.33	0.12	113.77	<0.0001
X_2_^2^	−0.92	0.12	55.01	0.0001
R^2^ = 0.96				

**Table 4:** Analysis of Variance (ANOVA) for response surface optimization using ethanol as solvent.

Term	Sum of Square	Degree of Freedom	Mean Square	F-Value	*p*-Value
**Vanillin Concentration**					
Model	0.040	5	0.0080	26.31	0.0002
Residual	0.0021	7	0.0003		
Lack of fit	0.0007	3	0.0002	0.71	0.5947
Pure error	0.0014	4	0.0003		
Total	0.042	12			
**Vanillin Yield**					
Model	16.29	5	3.26	30.30	0.0001
Residual	0.75	7	0.11		
Lack of fit	0.36	3	0.12	1.25	0.4041
Pure error	0.39	4	0.097		
Total	17.04	12			

**Table 5:** Regression coefficient of polynomial function from all response of ethyl acetate.

Term	Coefficient	Standard Error	F-Value	*p*-Value
**Vanillin Concentration**				
Intercept	0.32	0.011		
X_1_	0.12	0.0090	189.47	<0.0001
X_2_	0.046	0.0090	26.10	0.0014
X_1_X_2_	0.026	0.013	4.22	0.0792
X_1_^2^	−0.062	0.009603	41.08	0.0004
X_2_^2^	−0.060	0.009603	38.46	0.0004
R^2^ = 0.9764				
**Vanillin Yield**				
Intercept	2.77	0.073		
X_1_	1.39	0.057	585.49	<0.0001
X_2_	0.37	0.057	41.54	0.0004
X_1_X_2_	0.34	0.081	17.36	0.0042
X_1_^2^	−0.44	0.062	51.37	0.0002
X_2_^2^	−0.54	0.062	75.99	<0.0001
R^2^ = 0.9908				

**Table 6:** Analysis of variance (ANOVA) for response surface optimization using ethyl acetate as solvent.

Term	Sum of Square	Degree of Freedom	Mean Square	F-Value	*p*-Value
**Vanillin Concentration**					
Model	0.19	5	0.037	58.03	<0.0001
Residual	0.0045	7	0.0006		
Lack of fit	0.0015	3	0.0005	0.65	0.6214
Pure error	0.0030	4	0.0007		
Total	0.19	12			
**Vanillin Yield**					
Model	20.00	5	4.00	151.48	<0.0001
Residual	0.18	7	0.026		
Lack of fit	0.15	3	0.048	4.91	0.0791
Pure error	0.039	4	0.0099		
Total	20.19	12			

**Table 7:** The optimization of vanillin concentration (%) and yield (μg g^−1^) using factor combination analysis using ethanol volume (mL) and extraction time (min).

Criteria	Parameter	Standard Prediction
Factor	Volume of ethanol (mL)	39.86
Factor	Extraction time (min)	247.98
Response	Vanilin concentration (%)	0.165
Response	Yield vanilin (μg g^−1^)	2.596
Desirability	-	0.848

**Table 8:** The optimization vanillin concentration (%) and yield (μg g^−1^) from factor combination analysis using ethyl acetate volume (mL) and extraction time (min).

Criteria	Parameter	Standard Prediction
Factor	Volume of ethyl acetate (mL)	60
Factor	Extraction time (min)	159.56
Response	Vanilin concentration (%)	0.408
Response	Yield vanilin (μg g^−1^)	3.957
Desirability	-	0.985

**Table 9:** Actual and code levels of fixed variables used in RSM for ethanol and ethyl acetate.

Fix Variable	Symbol	Level				
		−1.414	−1	0	+1	+1.414
Ethanol						
Solvent volume (mL)	X_1_	11.72	20	40	60	68.28
Time extraction (min)	X_2_	70.29	120	240	360	409.71
Ethyl acetate						
Solvent volume (mL)	X_1_	11.72	20	40	60	68.28
Time extraction (min)	X_2_	35.15	60	120	180	204.85
